# l-Argininium ethyl sulfate

**DOI:** 10.1107/S1600536808029954

**Published:** 2008-09-20

**Authors:** Harutyun A. Karapetyan

**Affiliations:** a Molecule Structure Research Center, National Academy of Sciences RA, Azatutyan ave. 26, 375014 Yerevan, Republic of Armenia

## Abstract

The title compound, C_6_H_15_N_4_O_2_^+^·C_2_H_5_O_4_S^−^, exhibits nonlinear optical properties. An extensive hydrogen-bonding network [N⋯O = 2.786 (4)–3.196 (5) Å] links cations and anions into a three-dimensional structure.

## Related literature

For crystal structures and nonlinear optical properties of related compounds, see: Monaco *et al.* (1987 ▶); Petrosyan *et al.* (2000 ▶). For details of the synthesis, see: Petrosyan (2005 ▶).
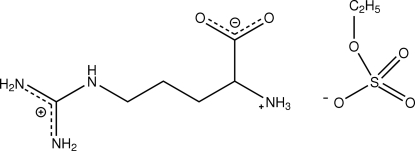


## Experimental

### 

#### Crystal data

C_6_H_15_N_4_O_2_^+^·C_2_H_5_O_4_S^−^
*M* *_r_* = 300.34
Orthorhombic, 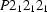

*a* = 9.1504 (18) Å
*b* = 12.519 (3) Å
*c* = 12.551 (3) Å
*V* = 1437.8 (5) Å^3^
*Z* = 4
Mo *K*α radiation
μ = 0.25 mm^−1^
*T* = 293 (2) K
0.26 × 0.22 × 0.14 mm

#### Data collection

Enraf–Nonius CAD-4 diffractometer
Absorption correction: none
4566 measured reflections
4171 independent reflections
3091 reflections with *I* > 2σ(*I*)
*R*_int_ = 0.061
3 standard reflections every 400 reflections intensity decay: none

#### Refinement

*R*[*F* ^2^ > 2σ(*F* ^2^)] = 0.071
*wR*(*F* ^2^) = 0.202
*S* = 1.03
4171 reflections
175 parameters
H-atom parameters constrained
Δρ_max_ = 0.74 e Å^−3^
Δρ_min_ = −0.59 e Å^−3^
Absolute structure: Flack (1983 ▶), 1775 Friedel pairs
Flack parameter: 0.05 (16)

### 

Data collection: *DATCOL* in *CAD-4 Manual* (Enraf–Nonius, 1988 ▶); cell refinement: *LS* in *CAD-4 Manual*; data reduction: *HELENA* (Spek, 1997 ▶); program(s) used to solve structure: *SHELXS97* (Sheldrick, 2008 ▶); program(s) used to refine structure: *SHELXL97* (Sheldrick, 2008 ▶); molecular graphics: *SHELXTL* (Sheldrick, 2008 ▶); software used to prepare material for publication: *SHELXTL*.

## Supplementary Material

Crystal structure: contains datablocks global, I. DOI: 10.1107/S1600536808029954/cv2443sup1.cif

Structure factors: contains datablocks I. DOI: 10.1107/S1600536808029954/cv2443Isup2.hkl

Additional supplementary materials:  crystallographic information; 3D view; checkCIF report

## Figures and Tables

**Table 1:** Hydrogen-bond geometry (Å, °)

*D*—H⋯*A*	*D*—H	H⋯*A*	*D*⋯*A*	*D*—H⋯*A*
N1—H1*B*⋯O5	0.89	1.94	2.823 (5)	173
N1—H1*C*⋯O6^i^	0.89	2.01	2.896 (4)	172
N1—H1*A*⋯O1^ii^	0.89	1.97	2.786 (4)	152
N2—H2⋯O3^iii^	0.86	2.25	3.098 (6)	170
N3—H3*B*⋯O6^iii^	0.86	2.35	3.196 (5)	167
N3—H3*A*⋯O2^iv^	0.86	1.93	2.771 (4)	165
N4—H4*B*⋯O1^iv^	0.86	2.00	2.847 (4)	170
N4—H4*A*⋯O4^ii^	0.86	2.11	2.945 (5)	165

Symmetry codes: (i) 
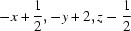
; (ii) 
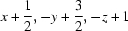
; (iii) 
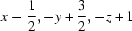
; (iv) 
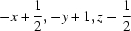
.

**Fig. 1.**
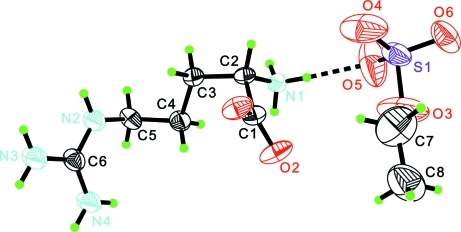
A perspective view of the asymmetric unit of (I) showing the atomic numbering and displacement ellipsoids at the 50% probability level.

## supplementary crystallographic information

### Comment

In a search of analogs of the L-arginine phosphate (LAP) a large number of new materials [Monaco *et al.*, 1987, Petrosyan *et al.*, 2000] have been obtained by the interaction of L-arginine with various acids by choosing appropriate conditions. The crystals from the interaction of L-arginine with H_2_SO_4_ could not be obtained due to extremely high solubility of reaction product (Petrosyan, 2005). Nevertheless, the conditions for obtaining the crystals of L-arginine salt with ethylsulforic acid were found (Petrosyan, 2005).

We present herein a structural study of the L-argininium ethylsulfate, C_6_H_15_N_4_O_2_^+^.C_2_H_5_O_4_S^-^, (I). A view of the asymmetric unit is shown in Fig. 1. The geometric parameters found in (I) are in a good agreement with the common accepted values. In the crystal, all eight active H atoms are involved in hydrogen bonding (Table 1), which link the kations and anions into three-dimensional structure.

### Experimental

The single crystals of (I) were obtained by slow evaporation of the aqueous solution of exchange reaction product described by Petrosyan (2005):

L-Arg × HBF_4_ + KC_2_H_5_SO_4_→ L-Arg × HC_2_H_5_SO_4_ + KBF_4_.

### Refinement

All H atoms were placed in geometrically calculated positions (C—H 0.96-0.98 Å, N—H 0.86-0.89 Å) and included in the refinement in a riding model approximation, with *U*_iso_(H)= 1.5*U*_eq_ (of Me- and N^+^H_3_ groups) and 1.2*U*_eq_ (other carrier atoms). High values of *U*_eq_ of some ethylsulforic anion atoms, except S, as compared to the other atoms of the structure, demonstrate potential thermal motion (rotation) of this group around the relatively heavy S atom.

### Crystal data

**Table:**

C_6_H_15_N_4_O_2_^+^·C_2_H_5_O_4_S^−^	*F*(000) = 640
*M**_r_* = 300.34	*D*_x_ = 1.387 Mg m^−^^3^
Orthorhombic, *P*2_1_2_1_2_1_	Mo *K*α radiation, λ = 0.71073 Å
Hall symbol: P 2ac 2ab	Cell parameters from 24 reflections
*a* = 9.1504 (18) Å	θ = 14–16°
*b* = 12.519 (3) Å	µ = 0.25 mm^−^^1^
*c* = 12.551 (3) Å	*T* = 293 K
*V* = 1437.8 (5) Å^3^	Block, colourless
*Z* = 4	0.26 × 0.22 × 0.14 mm

### Data collection

**Table:**

Enraf–Nonius CAD-4 diffractometer	*R*_int_ = 0.062
Radiation source: fine-focus sealed tube	θ_max_ = 30.0°, θ_min_ = 2.3°
graphite	*h* = 0→12
ω/2θ scans	*k* = 0→17
4566 measured reflections	*l* = −17→17
4171 independent reflections	3 standard reflections every 400 reflections
3091 reflections with *I* > 2σ(*I*)	intensity decay: none

### Refinement

**Table:**

Refinement on *F*^2^	Hydrogen site location: inferred from neighbouring sites
Least-squares matrix: full	H-atom parameters constrained
*R*[*F*^2^ > 2σ(*F*^2^)] = 0.072	*w* = 1/[σ^2^(*F*_o_^2^) + (0.0927*P*)^2^ + 1.2922*P*] where *P* = (*F*_o_^2^ + 2*F*_c_^2^)/3
*wR*(*F*^2^) = 0.202	(Δ/σ)_max_ < 0.001
*S* = 1.03	Δρ_max_ = 0.74 e Å^−^^3^
4171 reflections	Δρ_min_ = −0.59 e Å^−^^3^
175 parameters	Extinction correction: SHELXL97 (Sheldrick, 2008), Fc^*^=kFc[1+0.001xFc^2^λ^3^/sin(2θ)]^-1/4^
0 restraints	Extinction coefficient: 0.007 (2)
Primary atom site location: structure-invariant direct methods	Absolute structure: Flack (1983), 1775 Friedel pairs
Secondary atom site location: difference Fourier map	Flack parameter: 0.05 (16)

### Special details

**Table:**

Geometry. All e.s.d.'s (except the e.s.d. in the dihedral angle between two l.s. planes) are estimated using the full covariance matrix. The cell e.s.d.'s are taken into account individually in the estimation of e.s.d.'s in distances, angles and torsion angles; correlations between e.s.d.'s in cell parameters are only used when they are defined by crystal symmetry. An approximate (isotropic) treatment of cell e.s.d.'s is used for estimating e.s.d.'s involving l.s. planes.
Refinement. Refinement of *F*^2^ against ALL reflections. The weighted *R*-factor *wR* and goodness of fit *S* are based on *F*^2^, conventional *R*-factors *R* are based on *F*, with *F* set to zero for negative *F*^2^. The threshold expression of *F*^2^ > σ(*F*^2^) is used only for calculating *R*-factors(gt) *etc*. and is not relevant to the choice of reflections for refinement. *R*-factors based on *F*^2^ are statistically about twice as large as those based on *F*, and *R*- factors based on ALL data will be even larger.

### Fractional atomic coordinates and isotropic or equivalent isotropic displacement parameters (Å^2^)

**Table:**

	*x*	*y*	*z*	*U*_iso_*/*U*_eq_	
S1	0.23564 (11)	0.86276 (9)	0.82316 (10)	0.0575 (3)	
O1	0.1060 (2)	0.6386 (2)	0.5342 (2)	0.0447 (6)	
O2	0.3427 (3)	0.6749 (2)	0.5555 (3)	0.0541 (8)	
O3	0.3324 (5)	0.7594 (3)	0.8318 (5)	0.126 (2)	
O4	0.0977 (4)	0.8395 (4)	0.7810 (5)	0.114 (2)	
O5	0.3241 (6)	0.9208 (6)	0.7447 (3)	0.126 (2)	
O6	0.2501 (5)	0.9140 (2)	0.9236 (3)	0.0755 (11)	
N1	0.3133 (3)	0.8825 (2)	0.5232 (2)	0.0331 (5)	
H1A	0.3969	0.8533	0.5017	0.050*	
H1B	0.3140	0.8890	0.5938	0.050*	
H1C	0.3032	0.9467	0.4937	0.050*	
N2	0.1657 (4)	0.7002 (3)	0.1525 (2)	0.0450 (7)	
H2	0.0751	0.7186	0.1518	0.054*	
N3	0.0934 (4)	0.5395 (3)	0.0841 (3)	0.0531 (9)	
H3A	0.1139	0.4759	0.0629	0.064*	
H3B	0.0051	0.5627	0.0803	0.064*	
N4	0.3325 (4)	0.5638 (3)	0.1274 (3)	0.0519 (9)	
H4A	0.4024	0.6035	0.1505	0.062*	
H4B	0.3503	0.4994	0.1074	0.062*	
C1	0.2151 (3)	0.6992 (3)	0.5313 (3)	0.0340 (6)	
C2	0.1889 (3)	0.8130 (2)	0.4905 (2)	0.0296 (6)	
H1	0.0993	0.8406	0.5233	0.036*	
C3	0.1696 (3)	0.8141 (3)	0.3697 (2)	0.0332 (6)	
H3C	0.0883	0.7678	0.3515	0.040*	
H3D	0.1437	0.8860	0.3479	0.040*	
C4	0.3030 (4)	0.7788 (3)	0.3057 (3)	0.0392 (7)	
H4C	0.3842	0.8262	0.3209	0.047*	
H4D	0.3309	0.7072	0.3272	0.047*	
C5	0.2713 (4)	0.7799 (3)	0.1865 (3)	0.0408 (7)	
H5A	0.2353	0.8501	0.1670	0.049*	
H5B	0.3621	0.7683	0.1483	0.049*	
C6	0.1980 (4)	0.6018 (3)	0.1226 (3)	0.0400 (8)	
C7	0.2636 (9)	0.6578 (5)	0.8418 (6)	0.100 (2)	
H7A	0.2037	0.6434	0.7797	0.120*	
H7B	0.2012	0.6567	0.9043	0.120*	
C8	0.3784 (9)	0.5765 (5)	0.8517 (5)	0.098 (2)	
H8A	0.4249	0.5665	0.7838	0.147*	
H8B	0.3359	0.5103	0.8747	0.147*	
H8C	0.4496	0.5996	0.9029	0.147*	

### Atomic displacement parameters (Å^2^)

**Table:**

	*U*^11^	*U*^22^	*U*^33^	*U*^12^	*U*^13^	*U*^23^
S1	0.0378 (5)	0.0573 (6)	0.0773 (7)	0.0002 (4)	0.0002 (4)	−0.0298 (5)
O1	0.0284 (11)	0.0372 (12)	0.0686 (17)	−0.0036 (10)	−0.0039 (11)	0.0095 (12)
O2	0.0285 (12)	0.0425 (14)	0.091 (2)	−0.0015 (10)	−0.0121 (13)	0.0221 (14)
O3	0.067 (2)	0.070 (3)	0.241 (7)	0.014 (2)	−0.018 (3)	−0.067 (4)
O4	0.0435 (17)	0.094 (3)	0.205 (6)	0.0008 (19)	−0.016 (3)	−0.055 (3)
O5	0.103 (3)	0.213 (6)	0.061 (2)	−0.050 (4)	0.017 (2)	−0.028 (3)
O6	0.121 (3)	0.0484 (15)	0.0577 (17)	0.006 (2)	0.017 (2)	−0.0105 (14)
N1	0.0310 (12)	0.0337 (13)	0.0347 (12)	0.0004 (10)	−0.0024 (10)	−0.0021 (10)
N2	0.0364 (15)	0.0459 (16)	0.0528 (17)	0.0022 (13)	0.0005 (13)	−0.0137 (14)
N3	0.0346 (15)	0.0485 (17)	0.076 (2)	−0.0008 (13)	−0.0013 (16)	−0.0241 (17)
N4	0.0336 (15)	0.0494 (18)	0.073 (2)	0.0035 (13)	−0.0012 (15)	−0.0241 (17)
C1	0.0281 (14)	0.0341 (14)	0.0396 (15)	0.0023 (12)	0.0025 (12)	0.0055 (12)
C2	0.0214 (11)	0.0297 (13)	0.0377 (15)	0.0021 (10)	0.0025 (11)	0.0017 (12)
C3	0.0285 (13)	0.0346 (15)	0.0364 (14)	0.0024 (12)	0.0006 (12)	0.0001 (12)
C4	0.0299 (15)	0.0458 (17)	0.0421 (17)	−0.0007 (13)	0.0029 (13)	−0.0074 (14)
C5	0.0439 (18)	0.0385 (16)	0.0401 (17)	−0.0027 (14)	0.0093 (15)	−0.0078 (13)
C6	0.0355 (17)	0.0437 (18)	0.0406 (17)	−0.0023 (14)	0.0059 (14)	−0.0087 (14)
C7	0.118 (6)	0.072 (4)	0.111 (5)	−0.010 (4)	0.000 (4)	0.032 (3)
C8	0.153 (7)	0.057 (3)	0.084 (4)	0.005 (4)	0.022 (4)	0.002 (3)

### Geometric parameters (Å, °)

**Table:**

S1—O4	1.399 (4)	N4—H4B	0.8600
S1—O6	1.421 (3)	C1—C2	1.533 (4)
S1—O5	1.468 (5)	C2—C3	1.527 (4)
S1—O3	1.571 (4)	C2—H1	0.9800
O1—C1	1.255 (4)	C3—C4	1.526 (4)
O2—C1	1.244 (4)	C3—H3C	0.9700
O3—C7	1.426 (7)	C3—H3D	0.9700
N1—C2	1.490 (4)	C4—C5	1.524 (5)
N1—H1A	0.8900	C4—H4C	0.9700
N1—H1B	0.8900	C4—H4D	0.9700
N1—H1C	0.8900	C5—H5A	0.9700
N2—C6	1.322 (5)	C5—H5B	0.9700
N2—C5	1.453 (5)	C7—C8	1.467 (9)
N2—H2	0.8600	C7—H7A	0.9700
N3—C6	1.326 (5)	C7—H7B	0.9700
N3—H3A	0.8600	C8—H8A	0.9600
N3—H3B	0.8600	C8—H8B	0.9600
N4—C6	1.321 (5)	C8—H8C	0.9600
N4—H4A	0.8600		
			
O4—S1—O6	120.9 (3)	C4—C3—H3C	108.4
O4—S1—O5	110.3 (3)	C2—C3—H3C	108.4
O6—S1—O5	108.7 (3)	C4—C3—H3D	108.4
O4—S1—O3	111.3 (3)	C2—C3—H3D	108.4
O6—S1—O3	105.0 (3)	H3C—C3—H3D	107.5
O5—S1—O3	98.2 (4)	C5—C4—C3	111.2 (3)
C7—O3—S1	119.5 (4)	C5—C4—H4C	109.4
C2—N1—H1A	109.5	C3—C4—H4C	109.4
C2—N1—H1B	109.5	C5—C4—H4D	109.4
H1A—N1—H1B	109.5	C3—C4—H4D	109.4
C2—N1—H1C	109.5	H4C—C4—H4D	108.0
H1A—N1—H1C	109.5	N2—C5—C4	114.1 (3)
H1B—N1—H1C	109.5	N2—C5—H5A	108.7
C6—N2—C5	125.1 (3)	C4—C5—H5A	108.7
C6—N2—H2	117.5	N2—C5—H5B	108.7
C5—N2—H2	117.5	C4—C5—H5B	108.7
C6—N3—H3A	120.0	H5A—C5—H5B	107.6
C6—N3—H3B	120.0	N4—C6—N2	122.1 (3)
H3A—N3—H3B	120.0	N4—C6—N3	118.5 (3)
C6—N4—H4A	120.0	N2—C6—N3	119.4 (3)
C6—N4—H4B	120.0	O3—C7—C8	108.1 (6)
H4A—N4—H4B	120.0	O3—C7—H7A	110.1
O2—C1—O1	126.3 (3)	C8—C7—H7A	110.1
O2—C1—C2	117.1 (3)	O3—C7—H7B	110.1
O1—C1—C2	116.6 (3)	C8—C7—H7B	110.1
N1—C2—C3	110.9 (2)	H7A—C7—H7B	108.4
N1—C2—C1	109.3 (2)	C7—C8—H8A	109.5
C3—C2—C1	111.0 (3)	C7—C8—H8B	109.5
N1—C2—H1	108.5	H8A—C8—H8B	109.5
C3—C2—H1	108.5	C7—C8—H8C	109.5
C1—C2—H1	108.5	H8A—C8—H8C	109.5
C4—C3—C2	115.3 (3)	H8B—C8—H8C	109.5
			
O4—S1—O3—C7	−25.9 (8)	C1—C2—C3—C4	−63.9 (3)
O6—S1—O3—C7	106.5 (6)	C2—C3—C4—C5	178.5 (3)
O5—S1—O3—C7	−141.5 (6)	C6—N2—C5—C4	−89.4 (4)
O2—C1—C2—N1	−19.4 (4)	C3—C4—C5—N2	−67.5 (4)
O1—C1—C2—N1	162.5 (3)	C5—N2—C6—N4	4.4 (6)
O2—C1—C2—C3	103.2 (4)	C5—N2—C6—N3	−173.9 (3)
O1—C1—C2—C3	−74.9 (4)	S1—O3—C7—C8	−178.3 (5)
N1—C2—C3—C4	57.9 (4)		

### Hydrogen-bond geometry (Å, °)

**Table:**

*D*—H···*A*	*D*—H	H···*A*	*D*···*A*	*D*—H···*A*
N1—H1B···O5	0.89	1.94	2.823 (5)	173.
N1—H1C···O6^i^	0.89	2.01	2.896 (4)	172.
N1—H1A···O1^ii^	0.89	1.97	2.786 (4)	152.
N2—H2···O3^iii^	0.86	2.25	3.098 (6)	170.
N3—H3B···O6^iii^	0.86	2.35	3.196 (5)	167.
N3—H3A···O2^iv^	0.86	1.93	2.771 (4)	165.
N4—H4B···O1^iv^	0.86	2.00	2.847 (4)	170.
N4—H4A···O4^ii^	0.86	2.11	2.945 (5)	165.

Symmetry codes:  (i) −*x*+1/2, −*y*+2, *z*−1/2; (ii) *x*+1/2, −*y*+3/2, −*z*+1; (iii) *x*−1/2, −*y*+3/2, −*z*+1; (iv) −*x*+1/2, −*y*+1, *z*−1/2.
